# Hints and Improvements in the Practice of Medicine and Surgery

**Published:** 1799-05

**Authors:** 


### HINTS AND IMPROVEMENTS

### IN THE PRACTICE OF

### MEDICINE AND SURGERY.

### *On the febrifuge Virtues of Lime, Magnefia, and alkaline Salts in Dyfentery, Yellow Fever, and Scarlatina Anginofa.*

FROM a letter of Dr. J. BARKER, dated Portland (Maine), America, May 30th, 1798, and inferted at full length in the '*Medical Repofitory*,' Vol. II. No. II. we are happy to communicate to our readers the following important facts :—

" From the middle of Auguft to the firft of October, an epidemic fever, attended with dyfentery, was very prevalent in feveral towns. Adults were more frequently feized with it than children.

" The difeafe was ufhered in with pain in the abdomen, frequent ftools, naufea or vomiting, chillinefs fucceeded by heat, and great proftration of ftrength. The ftools were deeply tinged with blood, and very fetid. The matter rejected from the ftomach was of a dark green colour ; its tafte is defcribed to be like that of tartar emetic. A confiderable fever and thirft conftantly attended. The pulfe was quick and weak, though, during the exacerbation, which happened about once in twelve hours, large and full. The violence of the difeafe continued from one to two weeks, according to the means ufed for its melioration. Thirty fevere cafes, befides feveral gentle ones, fell to my fhare. I loft only one adult, to whom I was called at a late period, and two children, where very little medicine could be adminiftered.

" The mode of practice which I purfued, was to cleanfe the ftomach with ipecacuanha, and the inteftines with rheum and fal abfinth. or fal cathar. with fal abfinth.—Lubricating oils and mucilages were occafionally employed, as alfo enemas. But the remedies which I depended on to counteract the noxious caufe, were *alkaline falts and earths*. My common prefcription was :—aq. calc. one pound, fal abfinth. two drachms ; the dofe from one to two ounces every hour, and in fome cafes every half hour or oftener, in an infufion of flor. chamœm. Befides this, I ufed tefta. magnes. or creta, frequently, from one to two ounces in twenty-four hours. Calcined oyfter-fhells were fometimes employed, from two fcruples to one drachm the dofe.

" This

"This fever generally fubfided in ten or twelve days, where alkaline remedies were employed; but where they were not ufed, it was frequently protracted to twenty and fometimes thirty or more days, and then the patients feldom recovered."

After having cited feveral fuccefsful cafes, Dr. Barker thus proceeds:—"The recovery of thefe patients ferved more fully to eftablifh the credit of a mode of practice, which had been judged by fome to border upon rafhnefs, viz. exhibiting alkaline falts and lime-water in fevers*. During the months of November, December, January, February, and March, the fever which ftill continued, was attended, in moft cafes, with a fcarlet efflorefcence and fore-throat. It prevailed in almoft every town in the county, and was mortal in many inftances.

"The difeafe was ufhered in with the ufual fymptoms of naufea or vomiting, chilly fits, fucceeded by heat, &c. The throat foon became inflamed, which in a fhort time put on a gangrenous hue, and the breath was very fetid. In fome cafes, the throat was fwelled to fuch a degree, both externally and internally, that deglutition and fpeech were almoft entirely prevented; but there was no uniformity in the fymptoms. In fome there was no fore-throat or eruption: in three cafes which I faw, the virus was turned upon the renal glands, producing bloody urine, mixed with fkinny filaments, and attended with great pain, heat, and anguifh. In others there was diftreffing pain in the bowels, and thirft for water.

"After cleanfing the ftomach and inteftines, my efforts were particularly directed to counteract the virus, or noxious caufe. To effectuate this important purpofe, alkaline remedies were liberally employed, and their good effects were very apparent.

"Befides thefe, oils and mucilages were ufed to advantage. The mouth and throat, when particularly affected, were gargled with lime-water. This was evidently beneficial. It was as congenial to the ulcers and fores, as it is to ulcerations upon the external furface. Epifpaftics were applied to the neck, with a view to unload the glands of accumulated poifon. The blifters produced a great difcharge, and were very fore, attended in many inftances with intolerable itching. A lotion of lime-water readily allayed this itching, and

* The saline mixture and spt. mind. have ever been considered as safe and efficacious medicines in fevers; but we never fuspected their efficacy depended upon any alkaline power that was exerted after saturation, and our ignorance in this refpect, we presume is not very singular.

*Note of the American Editors.*

and difpofed the fores to heal, which in fome cafes appeared gangrenous.—I was called to two cafes, which terminated fatally. Thefe were all that I loft, out of more than fifty, in this diftemper.

"In one of thefe fatal cafes, that of a child, four years of age, who died on the ninth day of the diforder, upon diffection, the infide of the ftomach was found red and inflamed; the texture of the villous membrane deftroyed, grumous blood appearing in its ftead. A black liquor was alfo contained in the ftomach, which was in a contracted ftate. No marks of inflammation appeared in the inteftines; they contained a yellowifh fluid, and were dif-tended with air. The omentum was confiderably wafted, and of a red colour. No other morbid affection could be difcovered.

"I diffected a woman, fome years fince, who died of a puerperal fever, and found the ftomach in a fimilar condition with the child infpected. I faw another puerperal fubject diffected, where the rectum was alfo corroded and partly deftroyed. I then accufed the *bile* of being the mifchievous caufe; but I now believe that the ravages made in the ftomach, inteftines, throat, or other parts, muft be imputed to the *feptic acid;* and I am fully perfuaded that a feafonable and liberal ufe of *alkalines,* in all thefe different fpecies of fever, are efpecially indicated. Indeed, I conceive no other medicine that can, with any meafurable degree of propriety, be denominated febrifuges.

"For nearly three years I have purfued this mode of practice in febrile difeafes, and have kept exact records of cafes; from which it appears, that *I have not loft a fingle patient,* where thefe remedies have been particularly employed *from the commencement of the fever.*

"I found that opium and fpirits could be borne only in very fmall quan-tities in thefe fevers, efpecially in the firft ftage; and when the morbid excitement was confiderable, they were evidently injurious. Wine was offenfive to moft ftomachs; and the bark was by no means congenial. Mild bitters in decoctions were generally agreeable. Rice aud Indian gruel were the moft grateful articles of diet."

In concluding this interefting paper, we cannot omit to mention that Dr. Barker promifes, "before long," to publifh a particular account of thefe febrile diftempers, as they have appeared in America, for a few years paft, together with fuch obfervations as may be productive of public advantage.

### *Electricity employed to discover and destroy the Tape Worm.*

IN our last Number, p. 186, we barely mentioned that Dr. FRICKE of Brunswick, had lately made several succesful experiments, not only to discover that unwelcome visitor the *tape worm*, but likewise to destroy and expel it, by the powerful aid of electricity. The proper application of the electric fluid, according to the account of Dr. F. almost instantaneously relieves the most violent symptoms, such as anguish, oppression, spasmodic stricture in the præcordia, &c. The manner of applying electricity, to individuals suspected to be harrassed by the tape worm, is as follows:

Dr. F. uses a conductor with a globe of two inches and a half in diameter, from which he causes the sparks to strike against a globe of an insulated *scintillometer:* these sparks he passes in different directions through the abdomen; but, at first, admits them only from three to four inches long. As soon, however, as the patient can conveniently bear this kind of vibration, sparks to the length of from ten to twelve inches are admitted; and the more powerful these are, the more speedy will be the relief.

Dr. Fricke's scintillometer consists of a metal cylinder, thirty inches long, which lies insulated upon a pedestal, in a horizontal direction; it is divided into inches, and may be slided backwards and forwards. On one extremity, this cylinder is provided with a brass globe, four inches in diameter, on which the sparks strike from the conductor: the other extremity is provided with a ring. To this ring he fastens a metal chain or wire, covered with silk, which is connected with an insulated director. Another director, likewise insulated, is added, by means of a chain to that part of the machine which performs the friction: and by these two directors, which, for the sake of conveniency, are conjoined like a pair of tongs, the passage of the sparks is constantly regulated, while the patient sits on a common chair.

As the symptoms of the tape worm usually begin with severe tension and oppression, about the region of the stomach, the first sparks are directed through the pit of the stomach, in a straight line towards the vertebræ. After several sparks have been administered, eructations frequently take place; the patient feels much relieved in that particular part, but generally perceives the motion of another part of the worm, in some other place. Thither the sparks are again directed, and the worm is incessantly pursued, until it can be distinctly felt by the patient like a heavy weight.

To a lady who had been much troubled with the tape worm, Dr. Fricke prescribed half a drachm of powder of jalap, and carefully applied the electricity electricity in the manner before described, during the operation of this remedy: in consequence of such treatment, the patient discharged a tape worm upwards of twenty yards long. With respect to the necessary precautions in the use of electricity, in general, we refer the Reader to IMHOF's Remarks upon this subject, an abstract view of which we have given in the first Number of this Journal, pp. 55 and 56.

---

### *On the Medicinal Properties of the Datura Strammonium.*

IN our first Number, page 84, we gave a cursory account of the extraordinary effects of the *thorn-apple* on the human body, when taken internally; as stated by Dr. DE WITT, physician in the city of Albany, America;—at present, we shall furnish the reader with the substance of the "*medical observations on the virtues and properties of the seeds of the Datura Strammonium,*" as described by another medical practitioner, Dr. ALEXANDER KING, of Suffield, Connecticut.

The seeds of this vegetable are, so far at least as Dr. King has made observations upon it, the only parts useful in the Materia Medica; as they contain all the virtues of the plant, refined and prepared by the hand of nature.

These seeds, by expression, yielded a mucilaginous oil, which, when externally applied, is cooling, anodyne, and repellent. The virtues are readily obtained by decoction, in an aqueous menstruum. Half a drachm of the bruised seeds, boiled in four ounces of water, until half the quantity be evaporated, is a suitable portion for an adult in twenty-four hours, to be taken in divided doses.

An extract may be obtained by boiling any quantity of the bruised seeds, in a convenient proportion of water, for the space of four hours; then strain off the liquor by pressure; evaporate it over a gentle fire, without taking off the scum, until it have acquired the thickness of a syrup; then remove the liquor from the fire, and place it in a warm oven, in an earthen glazed vessel, until the aqueous parts be evaporated, and it become of a proper consistence for use. The dose is from half a grain to one grain for an adult.

The operation of this medicine, when taken in small doses, is moderately diuretic, and impregnates the urine pretty sensibly with the smell of the seeds. It is cooling, anodyne, and sedative. It relaxes the tone of the solids, lessens the contractile force of the arterial system, and consequently moderates the violent attrition of the circulating fluids against their containing vessels, lowers lowers the pulſation of the arteries, and renders the pulſe ſlower, more uniform and equable, when excited by violent ſtimuli. If taken in large doſes, it produces the following ſymptoms: the firſt ſenſible effect is in the ſight; there appears a preternatural dilatation of the pupil of the eye; viſion is rendered indiſtinct and confuſed; objects appear multiplied, diverſified, and variouſly coloured; the patient complains that he cannot ſee clearly; he cannot diſcern a ſmall object, ſuch for inſtance as the point of a pin or needle; he ſees in the room objects which do not exiſt, and complains of a numbneſs of the head, attended with vertigo. It alſo affects the organs of ſpeech. He faulters in pronunciation; his tongue is rendered paralytic; or, when he attempts to put it out, he imitates the motions of a perſon who tries to do the ſame in a nervous fever. The whole nervous ſyſtem is diſordered; various parts of the body become paralytic. From the ſenſes it extends its influence to the mental faculties. The imagination is confuſed and diſturbed with fear. Terrifying apprehenſions perplex the mind, and impreſs on the countenance the image of this paſſion. Theſe ſymptoms all diſappear, without any medical aſſiſtance, in the ſpace of twenty-four or forty-eight hours, ſooner or later, according to the degree in which they prevailed.

Dr. King then relates two caſes of phrenitis, or rather inflammation of the meninges of the brain, in both of which he preſcribed the decoction prepared of the ſeeds of the datura, with uncommon ſucceſs; and, particularly in the ſecond caſe, he expreſſes himſelf in the following terms: "In this caſe I had a fair opportunity to obſerve the action of the medicine, *in an early ſtage of the diſeaſe*, which was cooling, anodyne, and ſedative." He alſo informs us, that he has uſed this decoction of the thorn-apple in two other caſes of the ſame diſorder, with equal ſucceſs; and, in one inſtance, without any advantage; but, in that caſe, a general torpor or inſenſibility had taken place, before he ſaw the patient, who conſequently died the next day.

This remedy has been further recommended as a remedy for epileptic fits; in which diſorder Dr. King has ſometimes preſcribed it, but without any apparent ſucceſs. According to his obſervation, the chalybeates afford a much more efficacious remedy in that diſorder.

### *Carbon, a new Remedy for habitual Coſtiveneſs.*

IN the '*Medical Repoſitory*,' conducted by Drs. MITCHILL and MILLER, we find, that ſimple carbon or charcoal, deprived of its oxygen by heat, has been adminiſtered in the New York Hoſpital, in fifteen or twenty caſes. In no inſtance has it failed to purge, and in ſeveral the purging and inteſtinal commotion commotion excited by it, have been fo great, that it became neceffary to difcontinue it. Combined, however, with carbonat of foda *(fal fodæ)*, and particularly with the addition of lenitive electuary, it has proved one of the gentleft and moft efficacious remedies for the removal of habitual coftivenefs, that is yet known. It is further ftated, that this combination has probably had other and more falutary effects, in a few cafes of fcrophula and confumption, in which it has been adminiftered. But further and more accurate obfervations are neceffary to determine this point.

The following formula is that ufed in the New York Hofpital. Take of lenitive electuary, four ounces; carbonate of foda, two drachms; and carbon, two drachms. Of this mixture, from half an ounce, to one and two ounces, are to be taken, twice, thrice, or oftener in the day, according to circumftances.

### *Hydrargyrum phofphoratum, a powerful new Remedy in the fecondary Stages of Syphilis.*

[Extracted from the 'Journal of Inventions of Gotha,' No. II.]

THE phofphoric acid enters into no combination with mercury in its metallic ftate. But if the mercury be previoufly diffolved in nitric acid, and if the phofphoric acid, in a liquid form, be dropped into that folution, a white precipitate is formed, which is a true metallic neutral falt, confifting of a combination of the phofphoric acid and mercury.

Although the phofphat of mercury has long been known as a chemical preparation, yet its practical application has been little, if at all, attempted. BERGMAN, in his phyfical and chemical tracts, gives the following directions for preparing this active medicine:—Eight ounces of vitriolic acid are carefully and well mixed with four pounds of water, in a capacious glafs veffel; to this mixture muft be gradually added fourteen ounces of white calcined bones, reduced to powder. The veffel ought then to be placed in a temperature of about 60° for three days, that it may gently digeft: during which time it ought to be frequently ftirred with a glafs rod. Then the whole fhould be filtered through fine linen, the fluid part faved in a feparate veffel, and the refiduum wafhed in diftilled water, till it be completely edulcorated. The liquor contains the phofphoric acid, feparated from the bones by means of the vitriolic acid, but in a ftate not entirely free from gypfum. This liquor muft be evaporated to drynefs, and the refiduum diffolved in the fmalleft poffible quantity of luke-warm water, when a confiderable portion of gypfum will remain undiffolved. After ftraining off all the liquor, it muft again again be diluted with diftilled water, and a folution of the pureft potafs added to it, until it be completely faturated. Thus the fmall portion of gypfum ftill held in folution will be decompofed, and fome calcareous earth be precipitated, which muft be feparated by filtration. The liquor then ought to be evaporated to a proper confiftence, and expofed in a cool place, for cryftallization. At firft appears a fmall portion of vitriolated tartar, which originates from the decompofition of the gypfum; but if the liquor be again evaporated, the phofphorated vegetable alkali will be produced in rhomboidal prifmatic cryftals. Thefe fhould be again diffolved in diftilled water, and afterwards decompofed by a fuperfaturated folution of mercury, in the nitric acid. The precipitate thus obtained, after having been completely edulcorated, by repeated affufions of warm diftilled water, fhould be flowly dried. By this procefs, if the directions given be properly attended to, the product will uniformly be the *pureft phofphat of mercury*.

This extremely active mercurial preparation, however, unlefs it be adminiftered with great caution, is very apt to produce naufea, violent vomiting, ptyalifm, and other difagreeable fymptoms, even when taken in dofes not exceeding half a grain.—With a view to avoid fuch accidents, it has been fuccefsfully prefcribed in the following formula:—Take of hydrargyrum phofphoratum, four grains; pulv. cort. cinnam. fourteen grains; facchar. alb. half a drachm; the whole is to be divided into eight equal parts, one of which is to be taken every morning and evening, unlefs falivation take place, when it ought to be difcontinued. Some patients, however, will bear from one to two grains of the phofphat of mercury, without inconvenience.

This remedy has been obferved to heal inveterate venereal ulcers in a very fhort time, nay in the courfe of a few days, particularly thofe about the pudenda. In venereal inflammations of the eyes, chancres, rheumatifms, and chronic eruptions, it has proved of eminent fervice. Upon the whole, if ufed with the neceffary precaution, and in the hands of a judicious practitioner, it is a medicine mild and gentle in its operation. The cafes, in which it deferves the preference over other mercurial preparations, are thefe: in an inveterate ftage of fyphilis, particularly in perfons of torpid, infenfible fibres—in cafes of exoftofis, as well as obftructions in the lymphatic fyftem—in chronic complaints of the fkin, &c. It is perhaps unneceffary to add, that thofe who would rather hunt after fpecifics, than ftudy the nature of difeafes, and the conftitutions of their patients, will be as frequently difappointed in the ufe of this, as in any other of their favourite remedies.

W.

### *A Propofal for a new Method of removing and curing Steatomatous Tumors.*

AS the extirpation of fteatomatous and other indolent tumors, by an operation with the knife, is frequently attended with difficulty and danger, on account of more or lefs violent hemorrhages attending fuch operations, any other practicable method of removing them would be at once acceptable and beneficial to the practice of furgery.

In the '*Gotha Journal of Inventions*', No, XII. we meet with an account given by Profeffor WIMMER, of the univerfity of Graetz, in Styria, relative to this fubject, which appears to deferve the ferious attention of every furgeon. The cafe related by the Profeffor is not a little extraordinary. The tumor was fituated on the neck of the patient, far exceeding the fize of the whole head, and amounting to the almoft incredible weight of from fixteen to eighteen pounds. Mr. Wimmer introduced a feton through the middle of this tumor, which, in the courfe of a few days, produced inflammation and confequent mortification; fo that large pieces of the fubftance of the mafs were gradually feparated, until the whole was entirely removed, and the patient completely recovered.

We regret that our limits do not admit of ftating the particulars of this interefting cafe, which the author has defcribed pathologically in a feparate pamphlet, in German, to which we refer the curious reader, and which is intitled, "*Hiftory and Cure of a remarkable fteatomatous Tumor on the Neck:* by J. WIMMER. 8vo. *Grätz*, with a plate."

In juftice, however, to the ingenious author, we fhall further remark, that befides the remarkable cafe before alluded to, he has treated *four* other patients afflicted with fteatomatous tumours in a fimilar manner, and with equal fuccefs. Hence he draws the following conclufion, "that in large fteatomatous tumors, which obvioufly contain fat, a mortification of this fubftance ought not to be confidered as an alarming fymptom, but rather as the moft certain and favourable termination of the complaint; provided the patient poffefs fufficient ftrength to undergo the natural procefs of fphacelous feparation, and that his fluids be not in a contaminated ftate".

Although we are not very fanguine in our expectations, that this practice will prove fuccefsful in every cafe of fteatomatous excrefcence, or that it would even be advifable to recommend that practice *indifcriminately*, in cafes of this nature, yet we confider Mr. WIMMER intitled to fome fhare of credit, for an attempt equally bold and ingenious.—If we were allowed to to ſuggeſt a remark, on this occaſion, it would not be ſuch as to induce practitioners raſhly to undertake this operation on tumours of this or a ſimilar kind.

As the vitriolic ether is, of all the fluid ſubſtances, the beſt calculated to produce the greateſt degree of cold during its evaporation, and as this extremely volatile agent has been obſerved to be of eminent ſervice in the reduction of encyſted hernia*, if carefully poured on the affected and ſurrounding parts of the rupture, it is not unreaſonable to conjecture, that a *timely* local application of the ether might be attended with equally happy effects in the removal of ſteatomatous tumors. We wiſh, however, that this ſuggeſtion may not be miſapplied, by inducing the inexperienced practitioner to trifle with ſo formidable a diſeaſe; as we are well perſuaded, that no external application will be powerful enough to cauſe the abſorption of a tumour which has *gradually* arrived at a conſiderable ſize.

* Vide "Dr. Duncan's Annals of Medicine", Vol. VII. for 1792.

